# A new erythroneurine leafhopper genus from Thailand (Hemiptera, Cicadellidae, Typhlocybinae), with description of three new species

**DOI:** 10.3897/zookeys.595.8159

**Published:** 2016-06-02

**Authors:** Yuehua Song, Zizhong Li, Christopher H. Dietrich

**Affiliations:** 1 School of Karst Science, Guizhou Normal University, Guiyang, Guizhou 550001, China; 2 Illinois Natural History Survey, Prairie Research Institute, University of Illinois, 1816 S. Oak St., Champaign, IL 61820, USA; 3 Institute of Entomology, Guizhou University, Guiyang, Guizhou 550025, China

**Keywords:** Homoptera, Auchenorrhyncha, morphology, taxonomy, new taxa

## Abstract

A new genus of tribe Erythroneurini from Thailand, *Thaioneura* **gen. n.**, including three new species: *Thaioneura nigrilinea* **sp. n.** (type species), *Thaioneura sinuata* **sp. n.** and *Thaioneura suphanburia* **sp. n.**, is described and illustrated and a key to species is provided. The new genus exhibits a pattern of interspecific variation in the hind wing venation that has not been observed in other genera of the tribe.

## Introduction

The tribe Erythroneurini Young (1952) is the largest tribe in the subfamily Typhlocybinae, comprising 193 genera and 1848 described species worldwide (Dmitriev 2016). The erythroneurine fauna of southeast Asia is particularly diverse, but many genera and species remain undescribed. Study of recently collected samples from Thailand revealed the presence of a new genus, *Thaioneura* gen. n., here established based on distinctive characteristics.

## Material and methods

Morphological terminology used in this work follows Dietrich (2005). Habitus photos were taken using a Canon EOS 5D Mark II camera and the Camlift V2.7.0 software. Multiple photographs of each specimen were compressed into final images with Zerene Stacker (64-bit) software. Body length was measured from the apex of vertex to the tip of forewings. Abdomens were removed from specimens and cleared in cold 10% KOH solution overnight. The cleared material was rinsed with water and stored in glycerine. An Olympus SZX12 dissecting microscope was used for specimen study and Olympus BX41 and BX53 stereoscopic microscopes were used alternately for drawing of the dissected male genitalia and wings. Holotypes of the new species are deposited at the Queen Sirikit Botanical Garden, Chiang Mai, Thailand and additional specimens examined are deposited at the Illinois Natural History Survey, Prairie Research Institute, University of Illinois at Urbana-Champaign, USA.

## Results

### Thaioneura gen. n.

Taxon classificationAnimaliaHemipteraCicadellidae

http://zoobank.org/696D483C-D605-4FB6-8B0F-2BD62C9C949F

#### Type species.

*Thaioneura nigrilinea* sp. n.

#### Description.

Vertex with single dark medial spot at apex. Forewing with symmetrical, multilobed, transcommissural brown longitudinal marking.

Head in dorsal view roundly produced, slightly longer medially than next to eye, wider than pronotum. Face with anteclypeus of male broader and more convex than that of female. Pronotum broad, moderately long, with posterior margin concave. Mesonotum with basal triangles and scutellar suture distinct. Forewing with inner apical cell wide, base oblique; outer apical cell very short; second apical cell widened distally; claval vein not delimited. Hind wing with CuA either confluent with MP for short distance and separate distally (Fig. 2), or completely confluent (Fig. 12), vein CuP connected to CuA or free.

**Figures 1–10.**
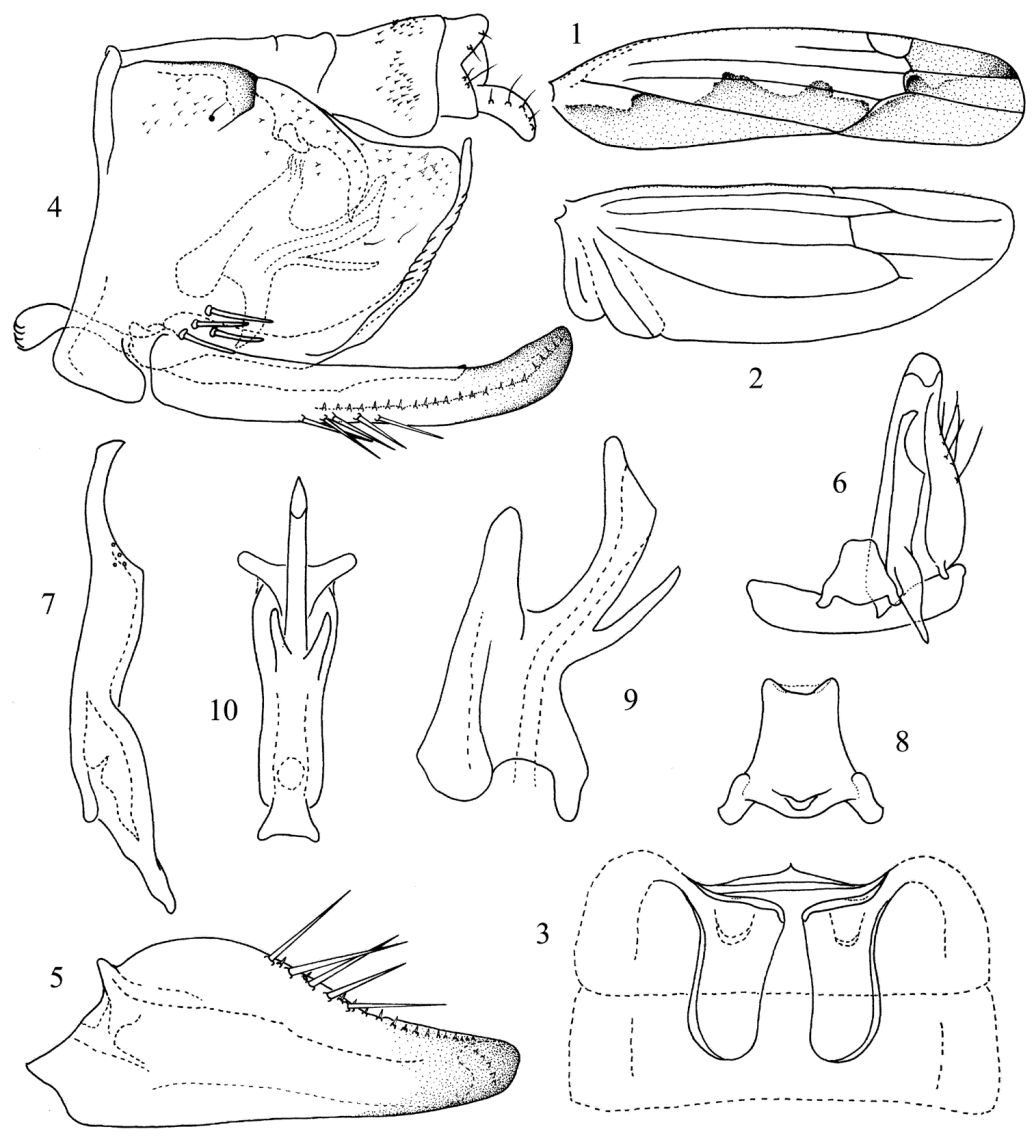
*Thaioneura nigrilinea* sp. n. **1** Forewing **2** Hind wing; **3** Abdominal apodemes **4** Genital capsule **5** Subgenital plate **6** Subgenital plate, Style, Connective and the 9th sternite **7** Style **8** Connective **9** Aedeagus, lateral view **10** Aedeagus, ventral view.

**Figures 11–20.**
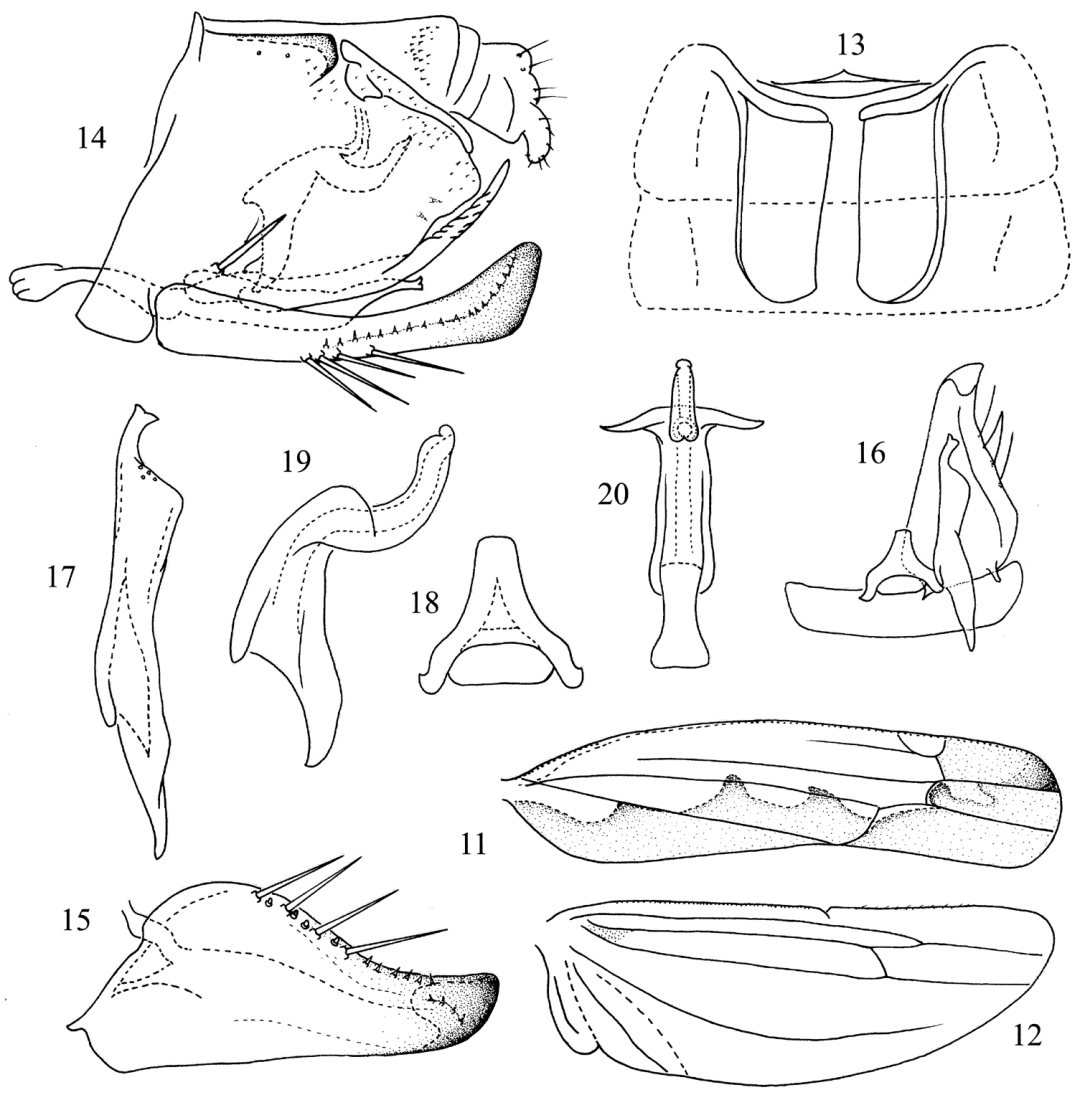
*Thaioneura sinuata* sp. n. **11** Forewing **12** Hind wing **13** Abdominal apodemes **14** Genital capsule **15** Subgenital plate **16** Subgenital plate, style, connective and the 9th sternite **17** Style **18** Connective **19** Aedeagus, lateral view **20** Aedeagus, ventral view.

Male 2S abdominal apodemes large, broad, extended to or beyond middle of 4th sternite.

Male genitalia. Male pygofer side with posterodorsal margin bluntly angulate, dorsal appendage movably articulated basally, tapered distally, not extended beyond pygofer apex; ventral appendage long, slender, rugose, extended along posteroventral margin of lobe to point slightly beyond posterodorsal apex of lobe; basolateral setae distinctly enlarged, long fine setae sparse; microtrichia near posterodorsal margin well developed. Subgenital plate narrow in lateral view, broad basally and tapered distally in ventral view, without angulate basolateral projection and stout basolateral setae, with 4–5 macrosetae near lateral margin medially and row of short rigid microsetae from middle to subapex, apex darkly pigmented. Style with apex truncate and slightly expanded, preapical lobe prominent but not acutely angulate, base slim in lateral view. Connective central lobe broad, lateral arms short, stem long. Aedeagus with dorsal apodeme expanded laterad, shaft arched near base in lateral view, gonopore terminal, on ventral surface.

#### Distribution.

Thailand.

#### Diagnosis.

The new genus is similar to *Balanda* Dworakowska, 1979 and *Tautoneura* Anufriev, 1969 in body shape, the presence of both dorsal and ventral appendages and a group of long stout basolateral macrosetae on the male pygofer, and the presence of a median anterior lobe on the connective, but differs in having the apex of the style truncate, the subgenital plate with reduced chaetotaxy basolaterally and the smoky brown commissural markings on the forewings. The latter color pattern, which is very unusual among Erythroneurini, closely resembles that of the type species of *Jalalia* Ahmed 1970, described from Pakistan, but that genus has the head narrower than the pronotum, lacks a ventral pygofer appendage, and has the style apex acuminate.

#### Etymology.

The new genus name was formed by combining the name of the country in which all known specimens were collected, “Thailand” with the common suffix for generic names in this tribe, “-neura”. The gender is feminine.

#### Key to species of *Thaioneura* (males)

**Table:** 

1	Aedeagal shaft with processes	**2**
–	Aedeagal shaft without process (Figs 19, 20)	***Thaioneura sinuata* sp. n.**
2	Aedeagal shaft with pair of sub-apical processes (Figs 29, 30)	***Thaioneura suphanburia* sp. n.**
–	Aedeagal shaft without sub-apical process (Figs 9, 10)	***Thaioneura nigrilinea* sp. n.**

**Figures 21–30.**
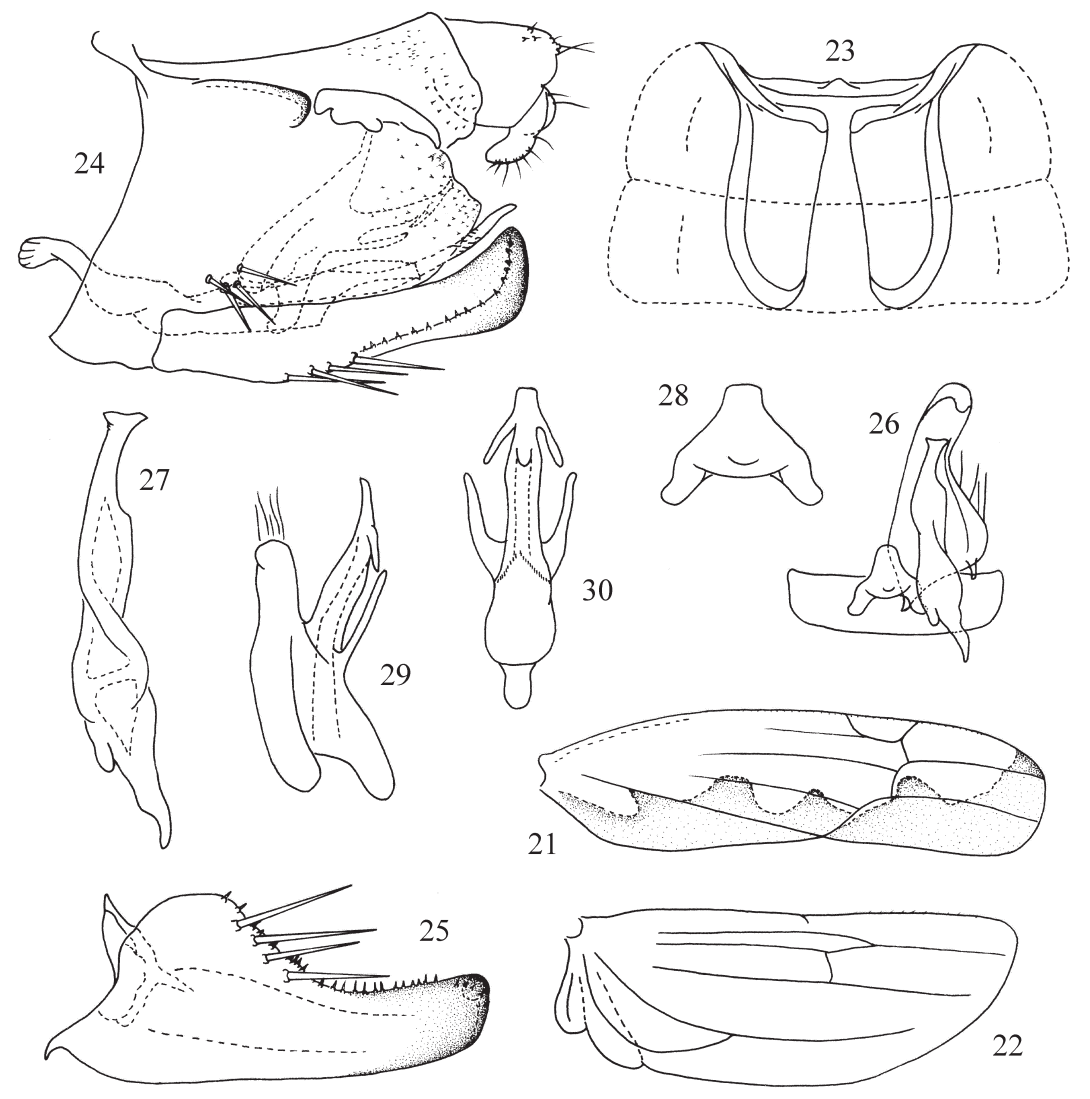
*Thaioneura suphanburia* sp. n. **21** Forewing **22** Hind wing **23** Abdominal apodemes **24** Genital capsule **25** Subgenital plate **26** Subgenital plate, style, connective and the 9th sternite **27** Style **28** Connective **29** Aedeagus, lateral view **30** Aedeagus, ventral view.

### Thaioneura nigrilinea sp. n.

Taxon classificationAnimaliaHemipteraCicadellidae

http://zoobank.org/DB18AD07-9EE5-4715-A726-C492BF2CD299

Figs 1–10 , 31A–D

#### Description.

Male length 2.3–2.4 mm.

**Figures 31.**
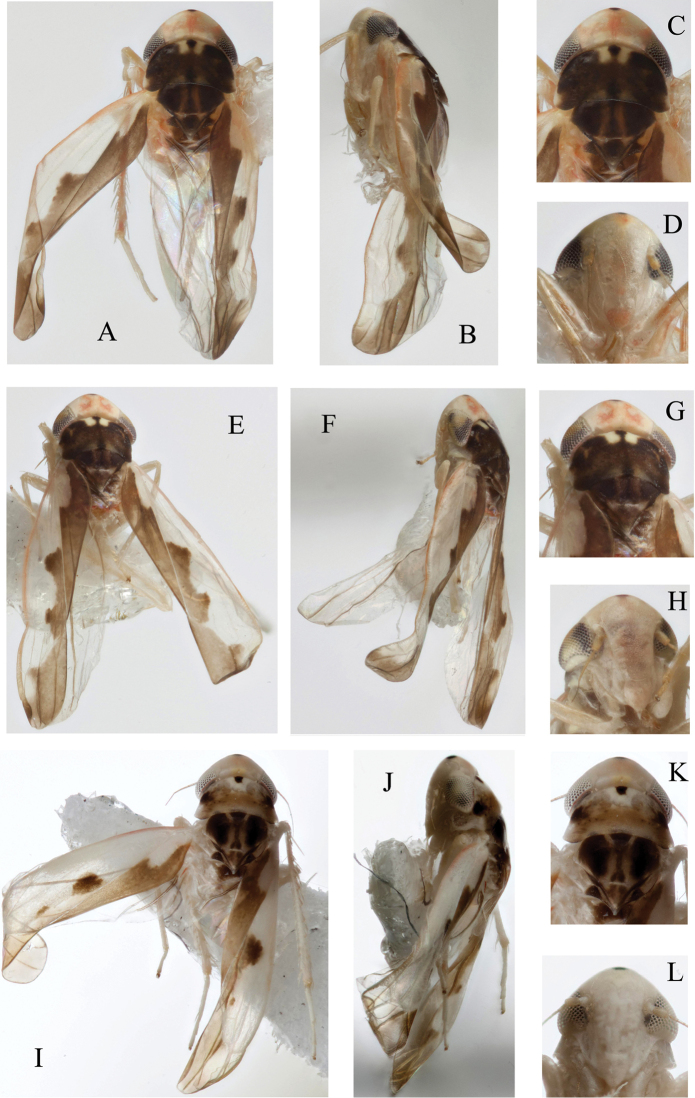
**A–D** *Thaioneura nigrilinea* sp. n. **E–H** *Thaioneura sinuata* sp. n. **I–L** *Thaioneura suphanburia* sp. n. **A, E, I** Habitus, dorsal view; **B, F, J** Habitus, lateral view **C, G, K** Head and thorax, dorsal view **D, H, L** Face.

Vertex milky yellow, with indistinct reddish cruciform mark medially; coronal suture weakly delimited (Fig. 31A, C). Face pale, anteclypeus with distal half reddish (Fig. 31D). Pronotum mostly dark brown with small pale submedial spots on anterior and posterior margins (Fig. 31A, C). Mesonotum brown, basal triangles and area between basal triangles dark brown; scutellum dark brown (Fig. 31A, C). Forewing with smoky brown markings as in Fig. 31A.

Hind wing vein CuA confluent with MP for short distance, then diverging and joining CuP near apex (Fig. 2).

Male abdominal apodemes extended to middle of 4th sternite (Fig. 3).

Pygofer side with dorsal appendage falcate; ventrolateral setal group with 4 macrosetae (Fig. 4). Subgenital plate with 5 marginal macrosetae (Figs 4, 5). Style apex slightly curved and obliquely truncate, preapical lobe sharply angulate (Figs 6, 7). Connective stem bilobed apically (Figs 6, 8). Aedeagal shaft compressed, curved dorsad distally, slightly expanded and footlike apically, with pair of slightly divergent fingerlike processes basally; preatrium short (Figs 9, 10).

#### Material examined.

Holotype: ♂, Thailand, Chaiyaphum, Tat Tone NP Dry Dipterocarp Forest, 15°59.037'N; 102°2.103'E, 250 m, Malaise trap, 21–28.vi.2006, coll. Lumyai Ittichan. Paratypes: 1♂, Thailand, Sakon Nakhon, Phu Phan NP, Behind national park office, 17°3.488'N;103°58.497'E, 318 m, Malaise trap, 5–11.i.2007, coll. Sailom Tongboonchai; 4♂♂, Thailand, Kanchanaburi, Khuean Srinagarindra NP, Behind tourist center, 14°38.155'N; 98°59.85'E, 210 m, Malaise trap, 11–18.ix.2008, coll. Chatchawan & Boonkam; 3♂♂, Thailand, Kanchanaburi, Khuean Srinagarindra NP, Huai Mae Kamint/50m/SW of Tourist center, 14°29.972'N; 98°53.035'E, Malaise trap, 18–25.ix.2008, coll. Somboon & Daorueng; 1♂, Thailand, Kanchanaburi, Khuean Srinagarindra NP, Huai Mae Kamint/Head Quarter, 14°38.123'N; 98°59.657'E, Malaise trap, 9–16.x.2008, coll. Somboon & Daorueng; 1♂, Thailand, Suphanburi, Pu Toei NP, Huai-Tapern/next to waterfall, 14°58.934'N; 99°19.31'E, Malaise trap, 14–21.xi.2008, coll. Wangkum P.

#### Remarks.

This species can be distinguished from the other species of this genus by the pair of fingerlike aedeagal processes, the short preatrium and the expanded apex of aedeagal shaft in lateral view (Figs 9, 10).

#### Etymology.

The specific name is derived from the Latin words “nigra” (black) and “linea” (line), referring to the brown longitudinal marking on the fore wing (Fig. 1).

### Thaioneura sinuata sp. n.

Taxon classificationAnimaliaHemipteraCicadellidae

http://zoobank.org/14CDF145-288F-447D-97AB-45BC7C70C4F6

Figs 11–20 , 31E–H

#### Description.

Male length 2.2–2.3 mm, female length 2.3–2.4 mm.

Color pattern very similar to that of *Thaioneura nigrilinea* (Fig. 31E–G), face with anteclypeus pale, without reddish color (Fig. 31H).

Hind wing vein CuA completely confluent with MP distally, CuP free distally (Fig. 12).

Male abdominal apodemes extended to hind margin of 4th sternite (Fig. 13).

Pygofer dorsal appendage slender, digitiform, only weakly curved ventrad, with one large basolateral macroseta (Fig. 14). Subgenital plate with 4 marginal macrosetae (Figs 14, 15). Style apex slightly curved and truncate with medial notch, preapical lobe bluntly angulate (Figs 16, 17). Connective stem narrow and truncate apically (Fig. 18). Aedeagal shaft tubular, curved dorsad, without processes, preatrium moderately developed (Figs 19, 20).

#### Material examined.

Holotype: ♂, Thailand, Sakon Nakhon, Phu Phan NP, Dry evergreen near house no.1567, 16°48.627'N; 103°53.511'E, 512 m, Malaise trap, 4–10.vi.2007, coll. Winlon Kongnara. Paratypes: 3♂♂, Thailand, Phetchabun, Khao Kho NP Mix deciduous, 16°39.589'N; 101°8.185'E, 168 m, Malaise trap, 5–12.i.2007, coll. Somchai Chachumnan & Saink Singtong; 6♂♂, Thailand, Suphanburi, Pu Toei NP Huai Mongpae/red road, 14°56.985'N; 99°26.78'E, 300 m, Malaise trap, 16-23.vii.2008, coll. Saunbua.L.; 5♂♂, Thailand, Kanchanaburi, Khuean Srinagarindra NP, Huai Mae Kamint/50m/SW of Tourist center, 14°29.972'N; 98°53.035'E, Malaise trap, 18–25.ix.2008, coll. Somboon & Daorueng; 3♂♂, Thailand, Chaiyaphum, Tat Tone NP Pha Eang waterfall, 15°57.24'N; 101°54.72'E, 301 m, Malaise trap, 12–19.iv.2007, coll. Tawit Jaruphan.

#### Remarks.

This species is similar to *Thaioneura nigrilinea* on external appearance and genital structures, but can be distinguished by the aedeagal shaft without processes, the longer preatrium (Figs 19, 20) and the more slender, less curved pygofer dorsal appendage (Fig. 14).

#### Etymology.

The specific name is derived from the Latin word “sinuate” (curved in and out), referring to the sinuate aedeagal shaft in lateral view (Fig. 19).

### Thaioneura suphanburia sp. n.

Taxon classificationAnimaliaHemipteraCicadellidae

http://zoobank.org/92CD2FEB-8875-497D-9248-12A3BD5074BA

Figs 21–30 , 31I–L

#### Description.

Male length 2.3 mm.

Color similar to other congeners (Fig. 31A–D; E–G). Vertex milky yellow, with longitudinal milky white bandlike stripe medially (Fig. 31I). Face with anteclypeus pale (Fig. 31L). Pronotum dark color faded, with three dark spots on anterior margin and both sides (Fig. 31I). Mesonotum light brown, basal triangles dark brown, with irregular dark marking in area between basal triangles; scutellum light brown (Fig. 31I).

Hind wing vein CuA completely confluent with MP distally, CuP free distally (Fig. 22).

Male abdominal apodemes extended to hind margin of 4th sternite (Fig. 23).

Pygofer dorsal appendage digitiform, but short; ventrolateral setal group with 4 macrosetae (Fig. 24). Subgenital plate with 4 marginal macrosetae (Figs 24, 25). Style apex slightly curved and truncate with medial notch, preapical lobe bluntly angulate (Figs 26, 27). Connective stem narrow and truncate apically (Fig. 28). Aedeagal shaft tubular, truncate apically in ventral view, with pair of long slender divergent processes arising near base and extended distad, pair of shorter apical processes extended basolaterad, preatrium short (Figs 29, 30).

#### Material examined.

Holotype: ♂, Thailand, Suphanburi, Pu Toei NP Phu Toei hill top/road, 14°57.32'N; 99°26.972'E, 650 m, Malaise trap, 24–31.viii.2008, coll. Saunbua. L. Paratype: 1♂, same data as holotype.

#### Remarks.

This species can be distinguished from the other species of this genus by the two pairs of aedeagal processes, the short preatrium and the truncate apex of the aedeagal shaft in ventral view (Figs 29, 30).

#### Etymology.

This new species is named from the type locality, Suphanburi, Thailand.

## Discussion

Study of 31 leafhopper specimens representing 3 new species revealed that the new genus described here exhibits two different patterns of hind wing venation that are stable within species but variable between species. Hind wing vein CuA of *Thaioneura nigrilinea* separates from MP distally and is connected to CuP near the wing apex (Fig. 2). This is the usual venational pattern seen in the vast majority of Erythroneurini. However, the other two new species (*Thaioneura sinuata*; *Thaioneura suphanburia*) have vein CuA of the hind wing completely confluent with MP distally and vein CuP free distally (Figs 12, 22). This latter pattern also occurs in the Oriental genera *Diomma* Motschulsky (see Chiang and Knight 1990) and *Watara* Dworakowska. The two known species of *Watara* show the pattern consistently but some species of *Diomma* have CuA completely confluent with MP while others have these two veins divergent near the wing apex. Therefore, variation in hind wing venation is known to occur but is rare in other genera of Erythroneurini. Despite the observed variation in hind wing venation, placement of the three new species described here into a single genus is strongly justified by the unique dorsal color pattern and combination of features of the male genitalia. Nevertheless, the particular pattern of variation exhibited among *Thaioneura* species is not known to occur in other erythroneurine genera and further collecting and morphological study is needed to determine whether such variation occurs in other genera. The type species of *Thaioneura*, *Thaioneura nigrilinea*, has the usual venational pattern found in other Erythroneurini and, therefore, presumably represents the plesiomorphic condition for the new genus while the other two species are more apomorphic. This hypothesis should be tested by future phylogenetic analyses of Erythroneurini.

## Supplementary Material

XML Treatment for Thaioneura

XML Treatment for Thaioneura nigrilinea

XML Treatment for Thaioneura sinuata

XML Treatment for Thaioneura suphanburia
